# Autologous graft-versus-host disease induction in advanced breast cancer: role of peripheral blood progenitor cells

**DOI:** 10.1054/bjoc.2000.1499

**Published:** 2000-11-22

**Authors:** E van der Wall, T Horn, E Bright, J-L Passos-Coehlo, S Bond, B Clarke, V Altomonte, K McIntyre, G Vogelsang, S J Noga, J M Davis, J Thomassen, K V Ohly, S M Lee, J Fetting, D K Armstrong, N E Davidson, A D Hess, M J Kennedy

**Affiliations:** Bunting-Blaustein Cancer Research Building, Baltimore, MD, 21231, USA

**Keywords:** breast cancer, autoreactive T-cells, cyclosporine, CLIP, MHC-class II, peripheral stem cells

## Abstract

The purpose of the present study was to investigate the impact of the use of peripheral blood progenitor cells (PBPCs) on the induction of autologous graft-versus-host disease (GVHD) in patients with advanced breast cancer. 14 women with stage IIIB and 36 women with stage IV breast cancer received cyclosporine (CsA) 2.5 mg kg–1 i.v. daily, d 0–28, and interferon-gamma (IFNg) 0.025 mg/m2 s.c. qod, d7–28, following PBPC-T ± bone marrow transplantation (BMT). Preceding high-dose chemotherapy consisted of cyclophosphamide 6 g/m2 and thiotepa 800 mg/m2. Histologically proven ≥grade II cutaneous GVHD was induced in18/50 (36%) of patients and was independent of the source of haematopoietic support. In vitro studies showed that post-transplant, 76% of patients had developed auto-cytotoxicity against their own pre-transplant PHA-lymphoblasts. A significant correlation between the occurrence of GVHD ≥grade II and cytolysis was observed in the NK cell-line K562 and the T47D breast cancer cell-line. With a median follow-up of 2½ years, the overall survival (OS) is 58%, the disease-free survival (DFS) 26%, both independent of the development of GVHD and similar to what has been observed in other studies on high-dose chemotherapy in advanced breast cancer. It therefore remains unclear whether the induction of autologous GVHD with the occurrence of auto-cytotoxic lymphocytes can result in an anti-tumour effect in this group of patients. © 2000 Cancer Research Campaign http://www.bjcancer.com


*British Journal of Cancer* (2000) **83**(11), 1405–1411
© 2000 Cancer Research Campaign
doi: 10.1054/ bjoc.2000.1499, available online at http://www.idealibrary.com on IDEAL®

### Autologous graft-versus-host disease induction in advanced breast cancer: role of peripheral blood progenitor cells

**E van der Wall, T Horn, E Bright, J-L Passos-Coehlo, S Bond, B Clarke, V Altomonte, K McIntyre, G Vogelsang, SJ Noga, JM Davis, J Thomassen, KV Ohly, SM Lee, J Fetting, DK Armstrong, NE Davidson, AD Hess and MJ Kennedy**

Bunting-Blaustein Cancer Research Building, Baltimore, MD 21231, USA

Summary The purpose of the present study was to investigate the impact of the use of peripheral blood progenitor cells (PBPCs) on the induction of autologous graft-versus-host disease (GVHD) in patients with advanced breast cancer. 14 women with stage IIIB and 36 women with stage IV breast cancer received cyclosporine (CsA) 2.5 mg kg–1 i.v. daily, d 0–28, and interferon-gamma (IFNg) 0.025 mg/m2 s.c. qod, d7–28, following PBPC-T ± bone marrow transplantation (BMT). Preceding high-dose chemotherapy consisted of cyclophosphamide 6 g/m2 and thiotepa 800 mg/m2. Histologically proven ≥grade II cutaneous GVHD was induced in18/50 (36%) of patients and was independent of the source of haematopoietic support. In vitro studies showed that post-transplant, 76% of patients had developed auto-cytotoxicity against their own pre-transplant PHA-lymphoblasts. A significant correlation between the occurrence of GVHD ≥grade II and cytolysis was observed in the NK cell-line K562 and the T47D breast cancer cell-line. With a median follow-up of 2 $\frac{1}{2}$ years, the overall survival (OS) is 58%, the disease-free survival (DFS) 26%, both independent of the development of GVHD and similar to what has been observed in other studies on high-dose chemotherapy in advanced breast cancer. It therefore remains unclear whether the induction of autologous GVHD with the occurrence of auto-cytotoxic lymphocytes can result in an anti-tumour effect in this group of patients. © 2000 Cancer Research Campaign http://www.bjcancer.com

**Keywords:** breast cancer; autoreactive T-cells; cyclosporine; CLIP; MHC-class II; peripheral stem cells

Due in part to the introduction of peripheral blood progenitor cells (PBPCs) as a source of haematopoietic support, administration of high-dose chemotherapy in the treatment of solid tumours has been increasingly studied in recent years. Uncontrolled phase I and II studies of high-dose chemotherapy in metastatic breast cancer have suggested a possible increase in the percentage of patients who achieved a durable complete remission (Antman et al, 1997; Basade and Gulati, 1998; Zujewski et al, 1998). So far, only one small randomized Phase III trial has been published to support this improvement (Bezwoda et al, 1995) but final results of currently ongoing phase III studies of high-dose chemotherapy in high-risk primary and metastatic breast cancer are eagerly awaited. Expectations are however that whereas a small proportion of patients with metastatic breast cancer may benefit from intensive treatment, the majority will have resistant disease and will continue to relapse rapidly. Immunomodulation strategies, which might be non-cross resistant with high-dose chemotherapy (Fuchs et al, 1995), could provide a way to overcome chemotherapy-resistant cell-clones. In patients with acute myeloid leukaemia, the occurrence of GVHD following allogeneic bone marrow transplantation has been associated with a prolonged disease-free survival whereas use of an autologous graft to avoid GVHD-related morbidity resulted in increased relapse rates (Weiden et al, 1980).

*Received 2 August 1999*
*Revised 29 May 2000*
*Accepted 15 August 2000*

*Correspondence to:* AD Hess

Administration of Cyclosporine, CsA, a drug that influences the redeveloping immune system following syngeneic or autologous BMT, induces an auto-aggression syndrome with pathology identical to allogeneic GVHD (Glazier et al, 1983; Cooper et al, 1991). In this respect, Cyclosporine, known as an effective immunosuppressive drug (Kahan, 1982) is the agent most extensively evaluated for the induction of autologous (in human) or syngeneic (in rat) GVHD. CsA which inhibits thymic-dependent clonal deletion induces release of $CD8^+$ autoreactive T-cells that promiscuously recognize MHC class II determinants on syngeneic and MHC disparate cells (Hess et al, 1990; Fischer et al, 1995; Ruvolo et al, 1995). Critical for the induction of this autoaggression syndrome is the elimination of a peripheral regulatory mechanism that controls the autoreactive T cells (Fischer et al, 1995). Elimination of the regulatory T cells by the preparative regimen provides a permissive environment for the autoreactive T cells to manifest autoaggression. The first clinical study to suggest an antitumour effect of CsA induced GVHD following autologous BMT (auBMT) in humans was in lymphoma patients (Jones et al, 1989). The occurrence of GVHD was found to be associated with the presence of autoreactive T-cells directed against MHC class II (HLA-DR) antigen. Since breast cancer cells have been reported to bear HLA-DR antigen (Bartek et al, 1987; Whitford et al, 1992), similar studies were initiated in women undergoing auBMT for metastatic breast cancer (Kennedy et al, 1993, 1994). Autologous GVHD could be induced concurrent with the development of $\alpha/\beta$ TcR-postive $CD8^+$ autoreactive effector cells in the peripheral blood (Hess et al, 1997).

The use of PBPCs following high-dose chemotherapy has accelerated haematopoietic recovery thereby decreasing the number of myelosuppression-related complications (Chao et al, 1993). Since a PBPC-graft contains significantly more T-cells when compared to bone marrow, transfer of these T cells might restore the peripheral auto-regulatory mechanisms that impair the auto-reactive T-cells and, as a consequence, the induction of GVHD could be significantly reduced.

The present study aimed to define the influence of stem cell grafts on PBPCs stem cell grafts on the induction of autologous GVHD following high-dose chemotherapy. After high-dose alkylator chemotherapy, patients with stage III or IV breast cancer received either PBPCs alone or PBPCs with auBMT for haematopoietic rescue. Subsequently, patients were given CsA and IFN-γ to induce autologous GVHD. The administered dosages of CsA and IFN-γ given were in accordance with the doses as defined in the previous phase I study (Kennedy et al, 1994). Induction of GVHD was determined by clinical evaluation, evaluation of skin biopsies and lymphocyte-cytotoxicity assays using autologous pre-transplant PHA-lymphoblasts and 2 different pre-defined cell-lines. The incidence and degree of GVHD was compared with that observed in a prior study in which patients underwent high-dose chemotherapy supported with bone marrow alone (Kennedy et al, 1994).

### MATERIALS AND METHODS

### *Patients*

Women between the ages of 18 and 60 years with a ECOG performance status of <2 with histologically documented breast cancer which had metastasized or was either inoperable or locally recurrent were eligible. A minimum of 2 cycles of standard dose systemic chemotherapy without evidence of progressive disease preceded study entry.

Any oestrogen or progesterone receptor level was allowed. The renal, cardiac, pulmonary and haematopoietic reserves had to be adequate to undergo high-dose alkylator therapy. All patients had bone marrow biopsies that were negative for tumour by routine histologic examination.

Conduct of the trial was approved by the Joint Committee on Clinical Investigation of the Johns Hopkins Hospital. All patients provided informed written consent.

### *High-dose chemotherapy and PBPC-T/BMT*

PBPCs were mobilized following the subcutaneous (s.c.) administration of G-CSF at 5 μg kg day$^{-1}$ for 5 consecutive days, starting at the day of the bone marrow harvest. On the 6th day a 6-hour long high-blood flow leukocytophoresis was performed using a COBE Spectra apheresis machine, as previously described (Passos-Coehlo et al, 1995). In the first 18 patients bone marrow ($3 \times 10^8$ cells) was also collected, 4–6 weeks after administration of the last cycle of standard dose chemotherapy, and routinely reinfused the day following PBPC-reinfusion. In the remaining patients, bone marrow was collected only when less than $3 \times 10^6$ CD34$^+$ PBPC kg$^{-1}$ were harvested, which occurred in 10 patients. PBPCs were reinfused on day –1, two days after the end of high-dose chemotherapy. Marrow, if administered, was reinfused on day 0.

With a minimum of at least 5 days after the last dose of G-CSF for PBPC-mobilization, patients began high-dose chemotherapy with cyclophosphamide (Cytoxan) 1.5 g/m$^2$/day, in 250 ml D5 by continuous infusion (CI) and thiotepa (N,N′N″-triethylenethiophosphoramide) 200 mg/m$^2$/day in 500 ml D5 CI, both for 4 days (days –8 to –4). The high-dose chemotherapy and supportive care treatment was administered as previously described (Kennedy et al, 1994).

### Autologous GVHD induction

Cyclosporine (CsA) administration at 2.5 mg kg$^{-1}$ ideal body-weight day$^{-1}$ i.v. over 4 hours in 2 divided doses was initiated on day 0, to be continued for 28 days. Patients who were discharged before day 28 received CsA orally at a dose 4 times the i.v. dose given in 2 divided doses while whole blood levels were monitored. The oral dosage was increased 25% if levels were below 200 ng ml$^{-1}$. CsA dosing was modified for renal toxicity as described previously (Kennedy et al, 1994).

IFN-γ (provided by: Cancer Therapy Evaluation Program, Division of Cancer Treatment, National Cancer Institute) was administered daily at a dose of 0.025 mg/m$^2$ s.c., starting at day +7 through day +28. The only dose modification of IFN-γ was complete discontinuation of therapy for reasons of toxicity or severe GVHD, i.e. stage III rash covering more than 50% of the patients body (Glucksberg et al, 1974) and a skin biopsy diagnostic of >grade II GVHD (Sale et al, 1977).

Treatment of GVHD was begun when patients developed symptomatic skin GVHD and consisted of topical steroids and systemic antihistamines followed by untimely discontinuation of CsA. If ineffective or in case of visceral GVHD systemic prednisone i.v. was initiated and readily tapered upon clinical improvement.

### Evaluation of toxicity and immune reactivity

Patients were examined daily for evidence of rash, jaundice, and diarrhoea. Daily monitoring of serum chemistries, complete blood cell count and stool volume was undertaken. In addition, the time to platelet and WBC recovery and the incidence of non-haematological toxicities were prospectively monitored in each patient. 4-mm punch biopsies of skin were performed before start of high-dose chemotherapy (day –9), and on days +14 and +35 and at any other time GVHD was clinically suspected. Each biopsy was examined under light microscopy in a blinded fashion by one of the investigators (TH) who was unaware of the patients' clinical status. Haematoxylin-stained sections were graded for GVHD by previous published criteria (Bauer et al, 1993; Horn et al, 1994).

50 millilitres of peripheral blood were collected prior to high-dose chemotherapy (HDC) and on days +5, +12, +19, +26 and +33 for analysis of lymphocyte-mediated cytotoxicity by utilizing the $^{51}$Cr release assays (Hess et al, 1997). Peripheral blood mononuclear cells were isolated by Ficoll-Hypaque density centrifugation, washed in RPMI-1640, and resuspended in complete culture medium consisting of RPMI-1640, 10% fetal calf serum, 1% glutamine, 100 u ml$^{-1}$ penicillin and 100 μg ml$^{-1}$ streptomycin.

The lymphocytes were quantitatively tested for NK-cell function using the K562 cell line while MHC class II specific lytic activity was assessed by evaluating the ability of the lymphocytes to lyse autologous phytohaemagglutinin (PHA) stimulated lymphoblasts, cryopreserved prior to transplant. In addition, the MHC class II positive breast cancer cell line T47D, pre-incubated for 1 hour at 4°C in normal mouse serum (0.1% in RPMI-1640) or murine monoclonal antibody to HLA-DR (1:50 dilution in RPMI-1640) were used as target cells. Based on previous studies of au BMT recipients not treated with CsA, and patients tested prior to

© 2000 Cancer Research Campaign transplant, greater than 3.5% lysis by the post-transplant lymphocytes was considered indicative of autocytolytic activity.

Target and K562 cells were washed in RPMI-1640 and labelled with 100–200 μCi of $^{51}$Cr for 18 hours whereas T47D cells were plated at 3000 cells well$^{-1}$ in 200 μl plates and pulsed with 1–2 μCi of $^{51}$Cr for 18 hours as previously described (Hess et al, 1997). All target cells subsequently were washed in RPMI-1640 and resuspended in complete culture media. Effector and target cells were incubated in or triplicate wells at ratios of 100:1, 50:1 and 25:1 in a total volume of 0.2 ml for 4 hours. Supernatants were analysed on a γ-Coulter (Miniaxi γ, Packard Instruments Company, Downers Grove, IL) where maximum and spontaneous $^{51}$Cr release was calculated according to the following formula:

% specific $^{51}$Cr release = (cpm experimental – cpm spontaneous) × 100/(cpm maximum – cpm spontaneous). The variability between replicate cultures oversized less than 10%.

### Statistical evaluation

The association between the induction of GVHD and each of the variables of interest was measured using chi-square and/or Fisher's exact tests. Univariate logistic regressions were used to obtain the odds ratio and to test the significance of the association between GVHD and each of the continuous variables.

To compare DFS and OS for the binary or categorical variables of interest, Kaplan–Meier estimates and graphs were obtained. Log-rank tests were performed to test the equality of the survival functions across the groups. For the continuous variables, Cox regression analyses were used to examine the association between DFS, OS and each of the variables presented.

### RESULTS

### Patient characteristics

From 2/25/1994 until 11/8/1995, 50 patients were enrolled in the protocol with a median time to follow-up (TTFup) of 29.4 months (range 1.3–47.7).

As shown in Table 1, when divided according to means of haematopoietic support, both groups of patients were equally matched with regard to pertinent patient characteristics, i.e. age, performance status, stage of disease and hormone-receptor status.

### GVHD

Induction of autologous GVHD, defined as having skin-biopsy grade II or more (Bauer et al, 1993; Horn et al, 1994), was observed in 18 patients (36%), equally distributed over both groups of haematopoietic support (Table 1). Predictive characteristics for the development of GVHD grade II could not be defined (Table 2).

Two patients, in whom skin-biopsies revealed GVHD grade II, were diagnosed with subclinical GVHD. In 8 patients, the symptoms of GVHD required treatment with systemic corticosteroids. All of these patients were diagnosed, usually in the 3rd–4th week following PBPC-T ± BMT, with a stage III skin-rash and were observed at histological examination to have grade II GVHD. The length of corticosteroid administration varied from 15 days to

**Table 1** Patient characteristics

| | All patients (*n* = 50) | PBPCS + BM (*n* = 28) | PBPCS (*n* = 22) |
|---|---|---|---|
| BX grade ≥2 (no.) % pos. | (18) 36 | (10) 36 | (8) 36 |
| AGE years | | | |
| Mean (range) | 45 (28–61) | 46 (28–61) | 46 (29–60) |
| PS (no.)% | | | |
| 0 | (34) 68 | (20) 71 | (14) 64 |
| 1 | (16) 32 | (8) 29 | (8) 36 |
| STAGE (no.)% | | | |
| III B | (15) 30 | (9) 32 | (6) 27 |
| IV | (35) 70 | (19) 68 | (16) 73 |
| Visceral: | (9) 26 | (5) 26 | (4) 25 |
| Bone: | (14) 40 | (8) 42 | (6) 37.5 |
| Locoregional: | (12) 34 | (6) 32 | (6) 37.5 |
| RECEPTOR (no.)%* | | | |
| ER + | (22) 44 | (10) 36 | (12) 54 |
| PR + | (20) 40 | (11) 39 | (9) 41 |
| Unknown (both) | (8) 16 | (5) 18 | (3) 14 |
| DFS (no.)% | (13) 26 | (7) 25 | (6) 27 |
| OS (no.)% | (29) 58 | (16) 57 | (13) 59 |
| DOD (no.)% | (20) 40# | (11) 39# | (9) 41 |
| TTprog months | | | |
| Median (range) | 17 (0.8–47.7) | 16.5 (0.8–47.7) | 18.2 (1.4–38.5) |
| TTFup months | | | |
| Median (range) | 29.4 (1.3–47.7) | 31.9 (1.3–47.7) | 29.1 (7.5–40) |

PBPCS = peripheral blood progenitor cell support, BM = bone marrow, ER = oestrogen receptor, PR = progesteron receptor, GVHD = autologous graft versus-host disease, BX = biopsy, TTFup = time to follow-up, TTprog = time to progression, DFS = disease free survival, OS = overall survival, DOD = dead of disease. *Positive = ≥10 fmol mg$^{-1}$ cytosolic protein. # 1 patient died of CMV-infection.

**Table 2** Patient characteristics according to GVHD-status

| | GVHD >1 (*n* = 18) | GVHD 0 +1 (*n* = 32) |
|---|---|---|
| NO. OF PBPC mean (range) | | |
| CD34 × $10^6$/kg | 6. (0.5–20.5) | 4.7 (1.0–16) |
| AGE mean (range) | 48 (38–59) | 44 (28–61) |
| PS (no.)% | | |
| 0 | (13) 72 | (19) 59 |
| 1 | (5) 28 | (13) 41 |
| STAGE (no.)% | | |
| IIIB | (4) 22 | (11) 34 |
| IV | (14) 78 | (21) 67 |
| TTFup months | | |
| Median (range) | 34.6 (6.9–47.6) | 29.1 (1.3–47.7) |
| DFS (no.) % | (3) 17 | (10) 31 |
| OS (no.) % | (11) 61 | (18) 56 |
| DOD (no.) % | (6) 30* | (14) 44 |
| TTprog months | | |
| Median (range) | 16.5 (3.7–47.6) | 17.2 (0.8–47.7) |
| WBC >1000 days | | |
| Median (range) | 12.5 (10–22) | 12 (8–24) |
| PLAT TI days | | |
| Median (range) | 9 (5–26) | 8 (5–39) |

GVHD = autologous graft-versus-host disease, PBPC = peripheral blood progenitor cells, PS = performance status, TTFup = time to follow-up, DFS = disease free survival, OS = overall survival, DOD = dead of disease, TTprog = time to progression, WBC >1000 = white blood cell count >1000 × $10^6$/L, plat TI = days to platelet transfusion independence. *1 patient died of CMV-pneumonia.

$2\frac{1}{2}$ months. In 2 of the patients with biopsy-proven grade II GVHD of the skin, intestinal GVHD developed. Presenting symptoms of frequent diarrhoea and abdominal cramping were rapidly resolved by the administration of methylprednisolone i.v. in one patient. However, in the second patient, apparent chronic GVHD developed with a persistent malabsorption diarrhoea, abdominal cramping and fluctuating periods of nausea and vomiting. A small bowel follow-through showed skip areas of fibrosis and stenosis consistent with a GVHD-like process, which was confirmed by histological examination. Before the diagnosis of intestinal GVHD was made, the patient had already received methylprednisolone i.v. for her clinical stage III, histological grade II cutaneous GVHD. Increasing the dose of systemic corticosteroids did not improve the abdominal complaints. FK 506 was added but had to be discontinued after 1 week because of associated fevers. Subsequently oral CsA, 2.25 mg b.i.d. was initiated which reduced the severity of the intestinal symptoms. Both the methylprednisolone and the CsA were continued until shortly before her death. The patient developed pneumonia, shortly after analysis of her serum had shown signs of an active CMV-infection. Despite vigorous and appropriate systemic therapy the patient died of respiratory failure.

In the 8 patients in whom systemic treatment with corticosteroids was required, the lymphocytotoxicity data were not indicative of a more serious course of the GVHD when compared to the results of all GVHD-patients (Table 3).

### *Lymphocytotoxicity analysis*

Lymphocytes from the patients were assessed temporarily post-transplant for their ability to lyse pre-transplant lymphoblasts and the breast cancer cell line T47D. NK activity was also assessed utilizing the NK target cell line, K562. Representative experiments evaluating lymphocytotoxicity for 4 patients are shown in Figures 1A, B, C, D demonstrating the temporal development of autocytolytic activity. Two of the patients developed auto-GVHD (A, C) while the other two did not (B, D). Autocytolytic activity developed while the patients were on CsA but did not correlate with the development of auto-GVHD. Figures 2 A, B presents the autocytolytic activity for patients who developed auto-GVHD (A) and for those who did not develop this syndrome. One apparent difference between groups is that the autocytolytic activity in the patients with auto-GVHD appeared to develop later and persisted. This lytic activity could be detected early in the patients without auto-GVHD but waned quickly. The composite data analysing maximum lytic activity and time to this event are summarized in Table 3. Autocytolytic activity could be detected in 76% of all patients. Although not significant, there was a difference in median percentage cytolytic activity against autologous PHA-lymphoblasts between the patients who had developed GVHD and those who had not, being higher in the first group. Of interest, the day to maximum autocytolytic activity was delayed for the patients with GVHD compared to those patients who did not develop this autoaggression syndrome. Lymphocytes from the tested post-transplant also developed the ability to effectively lyse the T47D, MHC class II positive breast cancer cell line. Previous studies demonstrate that recognition of this cell line and the autologous lymphoblasts in the auto GVHD setting is MHC class II-dependent since pretreatment of the target cells with monoclonal antibody to MHC class II but not MHC class I determinants blocked lysis (Hess et al, 1997). Interestingly, the cytotoxic activity against the T47D cell line by the post-transplant lymphocytes of the patients with cutaneous GVHD grade II was significantly enhanced when compared with

 *© 2000 Cancer Research Campaign*

**Table 3** Lymphocytotoxicity analysis for all patients (percentage cell-kill by post-transplant lymphocytes)

| | ALL (*n* = 50) | GVHD (*n* = 18) | No GVHD (*n* = 32) |
|---|---|---|---|
| PHA-lymphoblasts, median (range) | 8.4 (4–43.7) | 11.3 (0.8–25.2) | 6.6 (0.4–43.7) |
| Day max, median (range) | 13 (5–33) | 19 (5–33) | 12 (5–33) |
| Patients (*n*) | 49 | 17 | 32 |
| PO. Patients > 3.5% kill | 37 | 13 | 24 |
| T47D, median (range) | 20.1 (7.4–57.1) | 30.3 (7.4–53.7)** | 18.3 (8–57.1) |
| Day max, median (range) | 19 (5–33) | 19 (5–33) | 19 (5–33) |
| Patients (*n*) | 39 | 11 | 28 |
| K 562, median (range) | 56.5 (28.2–100) | 60.4 (42.4–76.2)* | 52.35 (28.2–100) |
| Day max, med (range) | 19 (5–33) | 26 (5–33) | 19 (5–34) |
| Patients (*n*) | 49 | 17 | 32 |

Wilcoxon: **P* = 0.0395; ***P* = 0.0236. Lytic activity in peripheral blood lymphocytes was assessed post high dose chemotherapy and PBPC rescue. Maximum lysis (at a 100:1 effector:target ratio) of autologous lymphoblasts, T47D cells and the NK target K562 cells and time to maximum lysis were assessed lysis autologous lymphoblasts exceeding 3.5% was considered for autocytolytic activity. This cutoff value was determined evaluating control (non-CsA) au BMT patients.

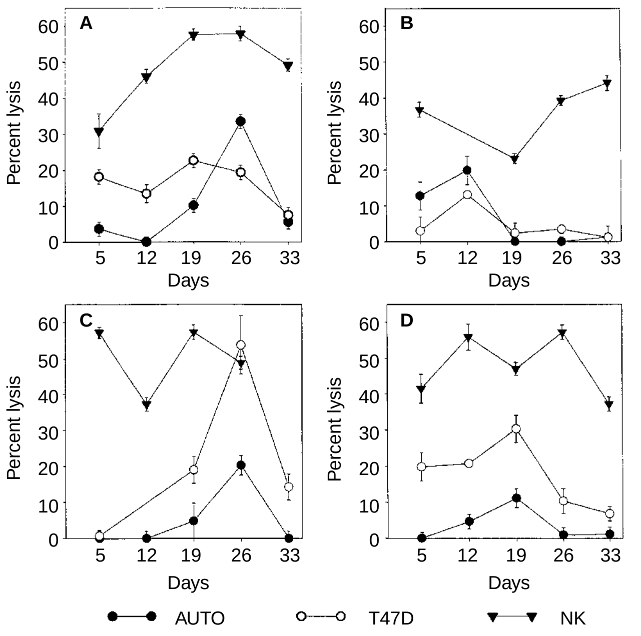

**Figure 1** Temporal assessment of lymphocytotoxicity post Au BMT and CsA treatment. Data (mean ± S.E.M.) represent lytic activity measured against autologous lymphoblasts, T47D cell line and the NK target, K562 at 100:1 effector:target ratio

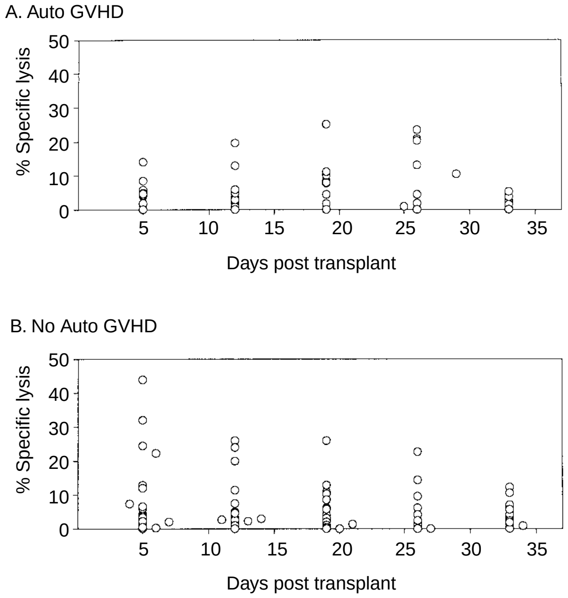

**Figure 2** Temporal assessment of autocytolytic activity for patients developing autologous GVHD (**A**) and for those who did not (**B**). Data represent lysis of pretransplant autologous lymphoblasts at a 100:1 effector:target ratio patients without GVHD (*P* = 0.0236). NK activity was also enhanced in this group of patients. If the lymphocytotoxicity values were taken as independent variables, only the percentage cell-kill of the T47D breast cancer cell-line was predictive of who would develop cutaneous GVHD grade II, *P* = 0.024 (data not shown).

### Survival data

With a median time to follow-up of 29.4 months (range 1.4–47.7) from time of PBPC-T ± BMT, the OS for all patients is 58%. The DFS is 26% whereas 16 patients (32%) are alive with disease with a median time to progression of 17 months (range 0.8–47.7) (Table 1). A number of well-known prognostic factors proved also to have a significant influence on the survival parameters in this study, i.e. stage of the disease, number of metastatic sites and skeletal involvement and whether patients received high-dose chemotherapy for recurrent disease or at first presentation. When patients were divided according to their GVHD-status, however, no significant differences could be demonstrated with regard to outcome (Table 2).

### Toxicity

CsA was discontinued early in 2 patients for reasons for nausea and vomiting in one and a change in liver-function in the second

© 2000 Cancer Research Campaign patient. In one patient a mild hypertension developed after administration of CsA, which was resolved by adding a calcium-channel blocker. Mucositis was diagnosed in 24/39 patients, and more than grade II in 10. One patient died of a (presumed) pulmonary CMV infection, as described earlier; otherwise, no serious infectious or septic episodes were recorded.

### DISCUSSION

Previous studies have shown that autologous GVHD could be induced in women with advanced breast cancer by the administration of high-dose chemotherapy followed by CsA (Kennedy et al, 1993) or CsA and IFNγ (Kennedy et al, 1994). In both studies, haematopoietic support was provided by autologous bone marrow. Moreover, the induction of this syndrome was associated with the development of $CD8^+$ autoreactive T cells that promiscuously recognize MHC class II antigens and could lyse MHC class II positive breast cancer cells (Hess et al, 1997). Promiscuous recognition of MHC class II was dependent on presentation of a peptide from the invariant chain peptide CLIP, with a superagonistic interaction between the N-terminal fragment of the peptide and the Vβ component of the T cell receptor (Hess et al, 1997, 1998). As PBPCs have replaced BM as the source of haematopoietic support, the current phase II study was designed to establish whether the use of PBPCs would affect the incidence of GVHD.

The results from the present studies suggest that there is a reduced incidence of autologous GVHD, defined as ≥grade II on skin biopsy (Bauer et al, 1993; Horn et al, 1994), in patients receiving PBPCs. In a previous phase I study, a 59% incidence of GVHD grade II was reported (Kennedy et al, 1994) compared to 36% observed in the present report. No difference in the induction of GVHD could be attributed to means of haematopoietic support, i.e. PBPCs alone or in combination with BM.

One possibility that might account for the decreased incidence of GVHD observed in the present study is the more strict definition of grade II histopathological cutaneous GVHD. In the present study, cutaneous GVHD ≥grade II required not only the presence of necrotic keratinocytes and satellite cell necrosis, as was used in the phase I study, but also significant lymphoid infiltration of the dermis. Dyskeratotic keratinocytes and satellite cell necrosis may be insufficient to make a histologic differentiation between eruptions of lymphocyte recovery and GVHD (Bauer et al, 1993; Horn et al, 1994).

On the other hand, PBPCs contain large number of T lymphocytes that may have regulatory potential. Studies in the animal of auto-GVHD indicate that transfer of even relatively small numbers of T lymphocytes was sufficient to inhibit the induction of this autoaggression syndrome (Fischer and Hess, 1990). Of additional interest in this regard is a recent report by Miura et al (2000). Autologous GVHD could not be induced in patients receiving PBPCs unless the T lymphocytes were removed. It is important to note, however, that the autoreactive T cells associated with this syndrome could be detected in both groups of patients even though there were no clinical manifestations of GVHD. Perhaps the T lymphocytes modified the pathogenic potential of the autoreactive T cells limiting their amplification and reducing the autoaggressive disease from a frank GVHD to a subclinical GVHD. It is clear in the animal system that these autoreactive T lymphocytes are the pathogenic T cells that manifest epidermal changes consistent with GVHD but are exquisitely regulated (Hess et al, 2000). Analysis of the lymphocytotoxicity profiles showed that the majority of the patients (76%), independent of their GVHD status, developed post-transplant lymphocytes capable of inducing lysis of pre-transplant PHA-lymphoblasts and is in accord with the results of Miura et al (2000). NK activity was also increased in patients who developed cutaneous grade II autologous GVHD. An increase in NK-cells has been reported in the occurrence of GVHD (Ghayur et al, 1987). Of potential importance in this study is the enhanced targeting of the T47D breast cancer cell line that may provide for antitumour immune activity. This enhanced targeting might be simply explained by the fact that this cell line may just be more susceptible to lysis. On the other hand, the T47D cells do express high levels of MHC class II-invariant chain peptide complexes leading to immune recognition by the $CD8^+$ autoreactive T cells (Hess et al, 1997).

In the previously reported phase I trial (Kennedy et al, 1994), with a median follow-up of nearly $7\frac{1}{2}$ years, the OS is 15% with a 6% DFS. With a median time to follow-up of $2\frac{1}{2}$ years in the present study, the OS is 58% with a 26% DFS, independent of the induction of cutaneous GVHD grade II. Considering the fact that the majority of the patients in the present study received treatment for stage IV disease, the survival percentages are certainly not less favourable than reported after high-dose chemotherapy alone (Antman et al, 1997). A possible explanation for the absence of a significant improvement in outcome might be that the autoreactive cytotoxic T-lymphocytes, while recognizing MHC class II determinants on a wide variety of tissues, do not preferentially target the tumour cells. This could limit the number of effector cells that can lyse the tumour cells. In addition, there may be insufficient amplification of the effector $CD8^+$-T cell population considering that regulatory T cells may be transferred with the PBPCs. Currently, the role of $CD34^\dagger$ stem cell selection (with the concurrent elimination of peripheral T cells) is under investigation.

In conclusion, administration of CsA and IFN-γ following high-dose chemotherapy in advanced breast cancer results in the induction of cutaneous GVHD grade II in a third of the patients without substantial increase of treatment-related morbidity. Autocytotoxic effector cells occur in the majority of patients and these cells may be equally important for an anti-tumour effect in a minimal residual disease setting or in a setting of very limited tumour burden. Therefore, in view of the fact minimal residual disease is the optimal condition to evaluate immunomodulation strategies, investigating the present treatment approach in the adjuvant setting seems appropriate.

### ACKNOWLEDGEMENTS

MJK was the recipient of an American Cancer Society Clinical Oncology Career Development Award. Supported by National Institutes of Health Grants No. A124319, CA 67800, CA15396.

### REFERENCES

Antman KH, Rowlings PA, Vaughan WP, Pelz CJ, Fay JW, Fields KK, Freytes CO, Gale RP, Hillner BE, Holland HK, Kennedy MJ, Klein JP, Lazarus HM, Mc Carthy PL, Saez R, Spitzer G, Stadtmauer EA, Williams SF, Wolff S, Sobocinski KA, Armitage JO and Horowitz MM (1997) High-dose chemotherapy with autologous hematopoietic stem-cell support for breast cancer in North America. *J Clin Oncol* **15**: 1870–1879

Bartek J, Petrek M, Vojtesek B, Bartkova J, Kovarik J and Rejthar A (1987) HLA-DR antigens on differentiating human mammary gland epithelium and breast tumours. *Br J Cancer* **56**: 727–733

*© 2000 Cancer Research Campaign*

Basade MM and Gulati SC (1998) High-dose chemotherapy in metastatic breast cancer. *Lancet* **351**: 386–387

Bauer DJ, Hood AF and Horn TD (1993) Histologic comparison of autologous graft-versus-host reaction and cutaneous eruption of lymphocyte recovery. *Arch Dermatology* **129**(7): 855–858

Bezwoda WR, Seymour L and Dansey RD (1995) High-dose chemotherapy with hematopoietic rescue as primary treatment for metastatic breast cancer: a randomized trial. *J Clin Oncol* **13**: 2483–2489

Busch R and Mellins ED (1996) Developing and shedding inhibitions: how MHC Class II molecules reach maturity. *Curr Opin Immunol* **8**: 51–58

Chao NJ, Schriber JR, Grimes K, Long GD, Negrin RS, Raimondi CM, Horning SJ, Brown SL, Miller L and Blume KG (1993) Granulocyte colony-stimulating factor “mobilized” peripheral blood progenitor cells accelerate granulocyte and platelet recovery after high-dose chemotherapy. *Blood* **81**: 2031–2035

Cooper MH, Hartman GG and Starz TE (1991) The induction of pseudo-graft-versus-host disease following syngeneic bone marrow transplantation using FK506. *Trans Proc* **23**: 3234–3235

Fischer AC and Hess AD (1990) Age related factors in Cyclosporine-induced syngeneic graft-vs-host disease: Regulatory role of marrow derived T lymphocytes. *J Exp Med* **172**: 85–94

Fischer AC, Ruvolo P and Burt R (1995) Characterization of the autoreactive T cell repertoire in cyclosporine-induced syngeneic graft-versus-host disease: a highly conserved repertoire mediates autoaggression. *J Immunol* **154**: 3713–3725

Fuchs EJ, Bedi A, Jones RJ and Hess AD (1995) Cytotoxic T cells overcome BRL-ABL-mediated resistance to apoptosis. *Cancer Res* **55**: 463–466

Ghayur T, Seemayer TA and Lapp WS (1987) Kinetics of natural killer cell cytotoxicity during the graft-versus-host reaction: Relationship between natural killer cell activity, T and B cell activity, and development of histopathological alterations. *Transplantation* **44**: 254–260

Glazier A, Tuschka PJ, Farmer ER and Santos GW (1983) Graft-versus-host disease in cyclosporin A treated rats after syngeneic and autologous bone marrow reconstitution. *J Exp Med* **158**: 1–8

Glucksberg H, Storb R and Fefer A (1974) Clinical manifestations of graft versus host disease in human recipients of marrow from HLA matched sibling donors. *Transplantation* **18**: 295–304

Hess AD, Fischer AC and Beschorner WE (1990) Effector mechanisms in cyclosporine A-induced syngeneic graft-versus-host disease. *J Immunol* **145**: 526–533

Hess AD, Bright EC, Thoburn C, Vogelsang GB, Jones RJ and Kennedy MJ (1997) Specificity of effector T lymphocytes in autologous graft-versus-host disease: role of the major histocompatibility complex class II invariant chain peptide. *Blood* **89**: 2203–2209

Hess A, Thoburn C, Bright E and Horwitz L (1998) Specificity of effector mechanisms in syngeneic graft-vs-host disease: recognition of the MHC class II invariant chain peptide (CLIP). *Transplant Proc* **29**: 725–727

Hess AD, Thoburn CJ, Chen W and Horwitz L (2000) Complexity of effector mechanisms in Cyclosporine-induced syngeneic graft-vs-host disease. Biology of Blood and Marrow. *Transplantation* **6**: 13–24

Horn TD, Bauer DJ, Vogelsang GB and Hess AD (1994) Reappraisal of histologic features of the acute cutaneous graft-versus-host reaction based on an allogeneic rodent model. *J Invest Dermato* **103**(2): 206–210

Jones RJ, Hess AD, Mann RB, Piantadosi S, Vogelsang GB, Farmer ER, Geller RB and Santos GW (1989) Induction of graft-versus-host disease after autologous bone marrow transplantation. *Lancet* **1**: 754–757

Kahan BD (1982) Cyclosporine. *N Engl J Med* **21**: 1725–1738,

Kennedy MJ, Vogelsang GB, Beveridge RA, Farmer ER, Altomonte V, Huelskamp AM and Davidson NE (1993) Phase I trial of intravenous cyclosporine to induce graft-versus-host disease in women undergoing autologous bone marrow transplantation for breast cancer. *J Clin Oncol* **11**: 478–484.

Kennedy MJ, Vogelsang GB, Jones RJ, Farmer ER, Hess AD, Altomonte V, Huelskamp AM and Davidson NE. (1994) Phase I trial of Interferon gamma to potentiate cyclosporine-induced graft-versus-host disease in women undergoing autologous bone marrow transplantation for breast cancer. *J Clin Oncol* **12**: 249–257

Miura Y, Nakao S, Veda M, Zeng W, Wang H, Takami A, Yamazaki H, Kawamura Y and Shiobara (2000). Autologous graft-versus-host disease with cyclosporin A after peripheral blood transplantation: Analysis of factors affecting induction. *J Allergy and Clin Immunol* (in press)

Ruvolo P, Bright E, Kennedy MJ, Morris LE, Fischer AC, Vogelsang GB, Jones RJ and Hess AD (1995) Cyclosporine-induced autologous graft versus host disease: assessment of cytolytic effector mechanisms and the Vβ T-cell receptor repertoire. *Transplant Proc* **27**: 1363–1365

Sale G, Lerner K, Barker E, Shulman HM and Thomas ED (1977) The skin biopsy in the diagnosis of acute graft-versus-host disease in man. *Am J Pathol* **89**: 621–625

Weiden PL, Sullivan KM, Flournoy N, Storb R and Thomas ED (1980) Antileukemic effects of graft-versus-host disease. Contribution to improved survival after allogeneic marrow transplantation. *N Engl J Med* **304**: 1529–1533

Whitford P, George WO and Campbell AM (1992) Flow cytometric analysis of tumor infiltrating lymphocyte activation and tumor cell MHC class I and II expression in breast cancer patients. *Cancer Lett* **61**: 157–164

Zujewski J, Nelson A and Abrams J (1998) Much ado about not enough data: high-dose chemotherapy with autologous stem cell rescue for breast cancer. *J Natl Cancer Inst* **90**: 200–209

*© 2000 Cancer Research Campaign*
